# Supplementary material for: Mentorship in health research institutions in Africa: A systematic review of approaches, benefits, successes, gaps and challenges
Source: PLOS Glob Public Health. 2024 Sep 23;4(9):e0003314. doi: 10.1371/journal.pgph.0003314 (PMC11419371; doi:10.1371/journal.pgph.0003314)
Supplement: S1 Table — (DOCX) [file pgph.0003314.s002.docx]

| **Participants -** Researchers at any career level, serving as mentors or mentees.  **Interventions -** Diverse mentoring programs of varied types, durations, and regularities  **Comparisons -** All mentorship programs were considered, regardless of the presence of a comparison group.  **Outcomes -** Mentorship approaches, benefits, successes, gaps, and challenges  **Settings -** African academic and/or research institutions | |
| --- | --- |
| **Database** | **Search strings** |
| **PubMed** | (Success OR Benefits OR Advantages OR Gaps OR Challenges) AND (mentor OR "mentorship practices" OR mentorship OR mentoring OR "mentoring relationship" OR "onsite training" OR "vertical mentorship" OR "on-the-job training" OR "capacity building" OR "capacity strengthening" OR mentee OR "mentoring program" OR "mentoring models" OR "career coaching" OR "career counselling" OR "career support" OR "mentorship advice") AND ("health research institutions" OR "Research institute" OR "health research centers" OR "research program" OR researchers OR "research organizations") AND (Africa OR African OR "sub-Saharan Africa" OR "Africa South of the Sahara" OR "East Africa" OR "West Africa" OR "Southern Africa" OR "Central Africa" OR "Northern Africa"). Filters: Books and Documents, Clinical Trial, Comparative Study, Evaluation Study, Guideline, Interview, Multicenter Study, Observational Study, Personal Narrative, Preprint, Randomized Controlled Trial, Technical Report, Validation Study, Humans, English |
| **Web of Science** | ALL=((Success OR Benefits OR Advantages OR Gaps OR Challenges) AND (mentor OR “mentorship practices” OR mentorship OR mentoring OR “mentoring relationship” OR “onsite training” OR “vertical mentorship” OR “on-the-job training” OR “capacity building” OR “capacity strengthening” OR mentee OR “mentoring program” OR “mentoring models” OR “career coaching” OR “career counselling” OR “career support” OR “mentorship advice”) AND (“health research institutions” OR “Research institute” OR “health research centers” OR “research program” OR researchers OR “research organizations”) AND (Africa OR African OR “sub-Saharan Africa” OR “Africa South of the Sahara” OR “East Africa” OR “West Africa” OR “Southern Africa” OR “Central Africa” OR “Northern Africa”)) and Review Articles (Exclude – Document Types) |
| **EMBASE** | (success OR benefits OR advantages OR gaps OR challenges) AND (mentor OR 'mentorship practices' OR mentorship OR mentoring OR 'mentoring relationship' OR 'onsite training' OR 'vertical mentorship' OR 'on-the-job training' OR 'capacity building' OR 'capacity strengthening' OR mentee OR 'mentoring program' OR 'mentoring models' OR 'career coaching' OR 'career counselling' OR 'career support' OR 'mentorship advice') AND ('health research institutions' OR 'research institute' OR 'health research centers' OR 'research program' OR researchers OR 'research organizations') AND (africa OR african OR 'sub-saharan africa' OR 'africa south of the sahara' OR 'east africa' OR 'west africa' OR 'southern africa' OR 'central africa' OR 'northern africa') |
| **AJOL** | *Mentorship |
| **JSTOR** | (((Success OR Benefits OR gaps OR challenges) AND (mentorship OR on-the-job training OR capacity building)) AND (Research institute OR health research centers)) AND (Africa) |
| **DOAJ** | Mentorship in health research in Africa (all fields) -<https://bit.ly/30tU37l> |
