# Supplementary material for: Mentorship in health research institutions in Africa: A systematic review of approaches, benefits, successes, gaps and challenges
Source: PLOS Glob Public Health. 2024 Sep 23;4(9):e0003314. doi: 10.1371/journal.pgph.0003314 (PMC11419371; doi:10.1371/journal.pgph.0003314)
Supplement: S2 Table — (DOCX) [file pgph.0003314.s003.docx]

| **Ref Id** | **Author, year, country, study design** | **Participants characteristics** | **Intervention Characteristics** | **Summary of key findings** | | | |
| --- | --- | --- | --- | --- | --- | --- | --- |
|  |  |  |  | **Gaps identified** | **Challenges** | **Benefits** | **Successes** |
| 1 | Daniels et al (2015) Kenya  Qualitative design | Early career researchers (n=152) involved in HIV/AIDS research, selected from a university | International HIV/AIDS research collaborations between investigators in the US and low-resource countries primarily designed to provide HIV/AIDS research training to Kenyan medical doctors and to non-medical trainees interested in an epidemiology or basic science research career (Socio-Behavioral Research, Biostatistics and Biomedical Research) at the University of Nairobi. The AIDS International Training and Research  Program (AITRP) follow up period was 10 years. | - Limited local research environment - Limited availability of funding - Limited availability of research career mentorship - Institutional demands on Kenyan faculty to teach rather than complete research restricts investigators' ability to develop research careers - The funding in Kenya among investigators is not at a level to support degree programs and post-doctoral training. without a solid financial foundation, those with advanced research training at the PhD level would leave for a supportive research environment | Training was not enough to ensure sustainable research careers.  Transition from trainee to early career and then to independent investigator takes time.  More senior investigators were needed to provide mentoring for early career investigators.  Inability to support early career investigators stemmed from a limited research culture in the Kenyan context Lack of local funding to support research Kenyans who reached independent investigator status had access to additional research funding, yet the ability for early career investigators to gain access to their own funding was limited without US collaborators. Long-term funding required for meaningful capacity strengthening is a major challenge.  Mismatch between the availability of short-term funding for specific research initiatives and the requirements for longer-term investment in capacity | International collaborations could provide that “fertile ground” during the transition as the Kenyan research environment continued to develop support for local investigators and research training in institutions. Pg 4 | - Mutually beneficial collaborations between Kenyan and US investigators developed during training built a supportive research environment for training.  - Early career investigators learned how to navigate the complex international research environment to build local HIV research capacity. - Shared and mutually beneficial resources within international research collaborations supported early career investigators and served as a conduit to transfer health research training to African institutions. - The study demonstrates the efficacy of capacity building models that harness international and local resources to support research careers |
| 2 | Ager & Zarowsky (2015) Southern, Eastern, Western Africa Qualitative design | Early career researchers (n=37) affiliated with a university, from the public health department | A collaborative capacity strengthening initiative delivered through international symposia, workshops, and research activities undertaken by the School of Public Health and Centre for Research in HIV and AIDS of the University of Western Cape (UWC), South Africa in collaboration with US based partners in HIV work. | - Establishing sustainable models of health research capacity development. Sources of financing are a central concern (particularly addressing the lack of host government investment), but also time scales in expectations of research activity.  - Lack of interest in investing in ‘novice’ researchers - Ensuring southern ownership: “how to support Southern-led priorities when much of the funding focus is Northern/ funder driven?”  - Weak South-South linkages (including lack of connection from Anglophone to Francophone and Lusophone contexts) and the loss of skilled re- searchers from the South (what one participant referred to as “the South-North research capacity strengthening initiative”). - Different interests of researchers, policymakers, and funders, and the difficulties in bridging between these agendas making the notion of ‘equitable partnership’ illusory.  - Lack of a clear national research strategy  - Frequent lack of clear policy demand from policymakers in the health sector | - Lack of attractiveness of research as a career in many Southern contexts. - Researchers are principally drawn by incentives ‘to consultancy not research’ - Securing long-term funding: Long-term funding required for meaningful capacity strengthening is a major challenge.  - Mismatch between the availability of short-term funding for specific research initiatives and the requirements for longer-term investment in capacity - Accommodating local health system priorities and constraints - Securing trust and cooperation - Finding common interest  - Addressing disincentives for academic engagement - Establishing and retaining research teams - Sustaining mentorship and institutional support | NS | NS |
| 3 | Daniels et al (2014) Kenya  Qualitative design | Early career health researchers (n=14), from a research institution involved in HIV/AIDS research | The study involved Kenyan HIV/AIDS researchers who had completed an international training program and had returned to Kenya after completing the program | - The study was conducted with Kenyan researchers and cannot be generalized to other global health programs in Kenya or African countries. | - Steep learning curve because of the difference in several aspects of education in Kenya and [the US].  - Access to journals was limited in Kenya for these participants.  - Most courses were taught from textbooks that are not always available. - - The individual perspective was that the move into first-world information and knowledge was a challenge.  - Tenuous research capacity among participants | - Eye-opener to international research - Led participants to perceive research to address HIV/AIDS in their community - Bridging of local knowledge with international knowledge in HIV/AIDS research fields while completing the curriculum in the training program. | Navigation of the learning curve during training, as demonstrated by the participants successful completion of/or progress in their training programs - Participants' willingness to develop their own research programs. - The program helped to contribute to more local knowledge. |
| 4 | Dartnall et al (2017) Tanzania, Kenya, Uganda Qualitative design | Early career researchers (n=14), recruited from a public health research institution | Sexual Violence Research Initiative: Participants were provided with intensive mentoring and technical advice in development or adaptation and conduct of preliminary proof of concept testing of violence against women and violence against children primary prevention interventions. Program duration = 3 years | small sample | - An uncertain and indirect route to research in Kenya - Research opportunities not readily available in Kenyan higher education | It provided partners with skills and confidence to advocate for the inclusion of primary prevention interventions for VAW and VAC in national violence prevention policies and ongoing programming plans. | Lessons learnt: Ongoing assessments are essential, self-transformation is imperative.  Mentorship through partnership, face-to-face meetings are invaluable for learning and inspiration, a systematic approach for long-term investment, developing shared measures and tools, value of south-south collaborations. |
| 5 | Balandya et al (2021) Tanzania Mixed-methods design | Early career researchers (n=63), from a public health teaching institution | Through the 5-year Transforming Health Professions Education in Tanzania (THET)-project, young peers received mentorship from senior researchers from a consortium through mentored research awards and research training, and in turn provided reciprocal peer-to-peer mentorship as well as mentorship to undergraduate students. | - Lack of protected time for research (this was a major bottleneck for both Young Peers and Senior Leaders at partnering institutions due to competing roles in teaching and administration) | - Delays in data collection, hiking up of prices and delays in delivery of procured research materials - Untoward effects of academic inbreeding, delays in disbursement of funds for project activities, amount of research funding disbursed to Young Peers was limited,  - Inability to hold physical meetings for research teams, quality of video conference sessions was sub-optimal during the first year of the project | - Fellows participated in all round training including in research training, in submitted grant applications; and making scientific presentations., courses undertaken in foundation course in epidemiology and biostatistics, sampling techniques, qualitative and quantitative research methodology, qualitative and quantitative data analysis, logistic regression, survival analysis in clinical research, research ethics, literature search, manuscript writing, grant writing, impact evaluation, result-based monitoring and evaluation, demographic and health survey, systematic review and meta-analysis, good clinical practice, introduction to reviewing genomic research as well as introduction to evidence-informed decision making - 6 Young Peers had registered in PhD programmes during the first two years of the project and 4 more were on course to register for PhD in project year 3 | - 1st two years of the project: three research articles published, two other manuscripts in final stages of preparation - The Young Peers shared authorship in one of the published articles.  - Most Young Peers had taken at least three research training short courses and six had enrolled in PhD programmes.  - Young Peers were beginning to broaden their research careers by involvement in other ongoing research projects and grant applications. |
| 6 | Ezeanolue et al (2019) Nigeria Qualitative design | Early career researchers (n=134) from a health research institution | Nigeria Implementation Science Alliance (NISA): Programme involved members of NISA involved on facilitating collaboration among partners, enhancing implementation research in Nigeria and the sub-Saharan region, and identifying feasible, culturally appropriate strategies to improve public health through research and participated in the 2017 NISA scientific conference. | - Poor mentorship - Inadequate training/lack of organized curriculum - Inaccessible opportunities - Lack of government funding - Lack of interest and motivation - Lack of research culture | - Research Assistants and Coordinators feeling overwhelmed because research is not their primary job - Research seems inaccessible especially to young people and outside academic settings - Inadequate funding - Defining qualification and selection process for research assistants/coordinators | Identified solutions to challenges:  - Increase opportunity to identify mentors (support conference attendance, like NISA) - Research module associated with institutions; create a forum for research related questions - Training/curriculum led by NISA, including appropriate research curriculum in educational settings - Increase government funding; advocate for higher appropriations for research, dedicate specific funds in grants for training research assistants - Increase pay for research assistants and coordinator positions to attract interest, create awareness of a potential career path for RA and RC positions, make training opportunities more flexible example: use online webinars - Use of social media to highlight research findings in Nigeria, more collaboration between research/academic institutions and non-academic/ research healthcare organizations | - Various organizations and government agencies have made definite commitments toward more investment in implementation research in Nigeria, For example, the National Agency for the Control of AIDS (NACA) in collaboration with the United Nations Children’s Fund (UNICEF) and the Population Council this year launched a pilot project called “Adolescent and Young People Challenge” that seeks to fund innovative ideas led by youth to provide comprehensive HIV education to at least 200,000 Nigerians, |
| 7 | Farnman et al (2016) Sweden, Uganda, South Africa, UK, Tanzania, Malawi, China, India, Finland, Oman, Vietnam Qualitative design | Mid-career researchers (n=16), from a health department in a university | ARCADE: 4-year North-South collaboration that used innovative educational technologies to strengthen health research across Africa and Asia focusing on postgraduate, doctoral and postdoctoral training. Cutting-edge online courses were developed, as well as blended learning modules and joint programmes that enabled training of researchers in LMICs who might not otherwise have access to such material. The program also worked at institutional level to strengthen education services, financial and administrative research management, research uptake capacity and network building. | NS | - Technical constraints and quality assurance - Adapting new teaching and learning methods into current university systems was challenging - Not being able to award students with credits for their degrees, the Asian consortium did not function as well - Some institutions in ARCADE RSDH found it difficult to reach out and establish new collaborations with other southern partners. | One of the main benefits of the ARCADE projects was the opportunity to establish new or improved collaborations between institutions. | The most successful form of collaboration was based around joint preparation of research proposals, and in that way exploring each other's areas of expertise, ARCADE projects have been successful in developing and delivering courses, and have reached over 920 postgraduate students |
| 8 | Gandhi et al (2019) Peru, Kenya, India, South Africa Qualitative design | Mid- or senior-level faculty researchers (n=132), teaching human health sciences, recruited from a university | A series of regional, 2-day intensive mentorship workshops were conducted for a period of 4 years to train mid- and senior-level investigators conducting public health, clinical, and basic science research across multiple academic institutions in LMICs to be more effective mentors. | - A lack of a culture of mentoring, time constraints, lack of formal training, and a lack of recognition for mentoring,  - Lack of an organized structure in mentoring relationships (including frequency of meetings, documentation of expectations, and goal monitoring), unclear expectations of a mentor–mentee relationship, discomfort with difficult conversations, and a lack of training on how to provide feedback.  - A lack of institutional structure to the mentoring process as a barrier to effective mentoring, with mentor–mentee relationships being created in an ad hoc manner without institutional oversight or attention to balancing the number of mentees per mentor in part because of a perceived lack of well-qualified mentors. | - Hierarchy, post-colonial legacy, and diversity,  - A lack of an institutional mentoring culture - Institutional failure to acknowledge or “give credit” for mentoring activities in the merit or promotions process - A general lack of time or time-management strategies to balance mentoring with other academic pursuits, and a lack of support for mentoring |  | These workshops provided valuable training, were among the first of their kind, were well-attended, rated highly, and provided concepts and a structure for the development and strengthening of formal mentoring programs across LMIC institutions, growth of institutional support and establishment of several new institutional mentorship training programs, and initiation of peer mentorship networks, regular mentor–mentee meetings, and IDPs. One newly established mentorship training model in Latin America was reported as being expanded as a national mandate for research training, nested within a required training program in the responsible conduct of research. |
| 9 | Gureje et al (2019) Ghana, Kenya, Liberia, Nigeria and South Africa Quantitative design | Mid-career researchers (n=35), from a research institution, involved in mental health research | A 4-year North-South multinational collaboration bringing together partner institutions from 5 African countries with researchers from USA and the UK in partnership with governments and NGOs to create an infrastructure to develop research capacity through workshops and fellowships targeting specific skill-sets as well as mentoring for early career researchers. | - Low policy priority afforded to mental health - Extreme paucity of human resources | - Carrying out intervention research in human subjects with severe mental disorders, human subjects who are prone to human right abuses - Forging partnerships among diverse stakeholders - Carrying out sustainable capacity building activities among scholars and other stakeholders from diverse regions on the continent | Early career and mid-level researchers from the five Sub-Saharan countries were first authors on 21 of the 60 published papers | -60 papers were published, and 21 successful grant applications made. establishment of a hub that evolves into a centre of research excellence with a crop of dedicated MH researchers, a sustainable partnership between researchers in SSA countries and other LMIC and with institutions in the north that facilitates collaborative cutting-edge research in global MH, as well as a management strategy that builds partnerships between local and international partners for efficient coordination and timely achievement of set goals- |
| 10 | Hakim et al (2018) Zimbabwe Mixed-method design | Early career researchers (n=166), from a medical school in a university | A 5-year medical education and research strengthening north-south collaborative initiative delivered through workshops facilitated by local and partner universities to offer a range of medical education and research capacity-focused programs including faculty development, research support, mentored scholars, visiting professors, community-based education, information and technology support, cross-cutting curricula, and collaboration with partner universities and the ministries of health and education. | -Uptake of technology-assisted learning and teaching has been slower than expected | NS | -Improved Internet connectivity and electronic resource availability,  -Full-time faculty grew by 36% (122 to 166), annual postgraduate and medical student enrollment increased by 61% (75 to 121) and 71% (123 to 210), respectively.  -To institutionalize and sustain MEPI innovations, the Research Support Center and the Department of Health Professions Education were established at UZCHS | -69% (115 of 166) of faculty members attended at least 1 of 15 faculty development workshops.  -Forty-one faculty members underwent 1-year advanced faculty development training in medical education and leadership. -Thirty-three mentored research scholars were trained under NECTAR, and 52 and 12 in cardiovascular and mental health programs, respectively |
| 11 | Somefun et al (2021) Uganda, Kenya, Tanzania, Nigeria, Malawi, South Africa Quantitative design | Early career researchers (n=143) comprising doctoral fellows in the health and social sciences field, recruited from universities and research institutions | Local research capacity strengthening programme delivered through PhD training fellowships. CARTA trains Africa-based doctoral fellows who admitted in cohorts with a goal of creating a network of locally trained but globally recognized scholars. | -Burdensome administrative roles -Inaccessibility of needed facilities  -Minimal opportunities to interact with young researchers -Lack of structured mentoring programme and unavailability of mentoring guidelines -Lack of funds to compensate for the time and effort that mentors commit to mentees. | -Limited time due to multiple roles, as a lecturer, doctoral student, and mentor, and administrative work,  -Attitude of mentees. Some fellows said that mentees sometimes lack confidence and have no interest in the field they are working in. Others seem unwilling to seek help. -Both mentors and mentees expressed frustration about lack of responsiveness. Mentors described mentees as being nonresponsive but equally mentees said that mentors did not always respond,  -Lack of mentoring skills, lack of motivation or zeal on the part of mentors and mentees, getting mentees to understand their roles, low achievement of set goals, and lack of or limited funding and resources. | NS | NS |
| 12 | Thombeka (2013) South Africa, Rwanda Qualitative design | Early career researchers (n=24), from a health research institution | Growing Researchers: Master’s and doctoral students were mentored by senior staff at two research councils: South African Medical Research Council (MR) and Human Sciences Research Council (HSRC) | -National priorities regarding scarce skills have a significant influence on the type of training which is prioritized by government and funders | -Students claim that research organizations get cheap labour through the internships, suggesting that the organization benefits at their expense, and that they, the students, do not benefit. -There is also the perception that the organizations succeed in meeting targets only regarding numbers of interns rather than making a meaningful contribution to the goal of increasing researchers. Interns apply for positions and are placed in units that may be far removed from their areas of specialization.  -Interns continue looking for permanent employment even as they start their internships, and as soon as they secure permanent employment, not necessarily in research or academia, leave the research council without completing the internship programme.  -The demands on the senior researchers’ time (the urgent need to obtain donor funds and report to donors, project managing, networking, and publishing attached to researchers’ performance appraisal) make the senior researchers’ work stressful and allow them little time to focus on interns who need significant guidance and advice. | -Most interns are involved in big and significant national and international research projects.  -Experience and demonstration of completeness makes them marketable as research practitioners.  -The interns who left the HSRC were offered attractive positions: one as a lecturer at a nearby university, another as a researcher in Parliament, and another as a director in a government department in the Northern Cape. | -From 2009-2010, 46 interns contributed to 39 peer-reviewed articles and in 2010/11,  -70 interns contributed to 51 peer-reviewed articles. |
| 13 | Thomson et al (2016) Rwanda  Quantitative design | Early career researchers (n=69), specifically statisticians recruited from a health research institution | A 6-week deliverable-driven survey analysis training based in Rwanda was conducted to strengthen skills of five local research leaders, 15 statisticians, and a PhD candidate |  | NS | -Model was effective in strengthening skills among full-time working professionals without disrupting ongoing work commitments and using few resources.  -Participants reported a high level of skill, knowledge and collaborator development from class-based training and out-of-class mentorship that were sustained 1 year later. | -Five of six manuscripts were authored by multi-institution teams and submitted to international peer-reviewed scientific journals, -Three-quarters of the participants mentored others in survey data analysis or conducted an additional survey analysis in the year following the training,  -In the 1 year following the training, 36% of participants completed an additional DHS analysis,  -71% completed an additional survey analysis,  -79% provided mentorship to others about survey data analysis. |
| 14 | Torondel, B et al (2019) Malawi, Kenya, Tanzania, Zambia, Ghana Quantitative design | Early career researchers (n=31), from a university in the sanitation and hygiene field | Sanitation and Hygiene Applied Research for Equity (SHARE): Delivered via (1) structured mentoring integrated into the research, administration, financial management and communication activities; (2) specific training to address immediate gaps in skills; and (3) a PhD programme designed to build lasting research capacity within LMIC institutions (including non-governmental organizations and universities) for 5 years | NS | -Different sets of administrative regulations across the institutions led to complications and delays in starting or sustaining certain capacity-building activities. -Difficulty in balancing work burden as mentees were involved in the programme research activities as well as the training and support for their institutions and their own development | NS | - 114 peer-reviewed manuscripts published in international journals - Formation of a peer network of researchers was the pivot of success of the SHARE programme – as a result nine networks were created during phase II of the project |
| 15 | Yukari C. Manabe (2018) Uganda Quantitative design | Early career health researchers (n=17) recruited from a university | MEPIMESAU: The program provided administrative support, paid tuition fees, tools (space, equipment, research money), skills (short research courses on study design, biostatistics, manuscript and grant writing), and infrastructure (finance, grants management support, and lab infrastructure) to early career researchers for 5 years | NS | - 2 (12%) PhD students withdrew from the doctoral training | - Strong outputs at lower cost and with relatively few additional mentors to rapidly achieve a critical mass of independent scientists able to conduct original research and mentor others. | -169 publications in peer-reviewed journals  -PhD students have supervised and mentored 65 master’s students,  -Additional 80 publications where MESAU fellows are co-authors that were not directly related to the PhD thesis work.  -The number of publications per student ranged widely (1–24) over the PhD period.  -Six of the PhD students have pursued research that has led to independent grant funding as well as collaborative grants on which they are listed as a co-investigator. |
| 16 | Lisa Langhaug (2020) Ethiopia, Malawi, South Africa, and Zimbabwe Qualitative design | Early career mental health researchers (n=52), recruited from a university | African Mental Health Research Initiative (AMARI): 48 researcher fellows at Master's PhD and post-doc levels were recruited and trained with the intent of equipping them with the necessary research, teaching, and leadership skills to build a viable and sustainable research network in the African region | -Stakeholders want high-impact African researchers, but few see a clear, replicable track for developing their careers within universities or their Ministries of Health in their African countries. | -Lack of support for infrastructure that enables high-quality research; including grants administration, mentorship, university leadership, research culture, and open communication between policymakers and researchers. | NS | NS |
| 17 | da Silva, Andrea Tenorio Correia (2019) Ethiopia, Malawi, South Africa, Uganda, and Zimbabwe, Ghana, Kenya, Liberia, Nigeria, and South Africa, and Sri Lanka Qualitative design | Early career mental health researchers (n=5), recruited from a research institution | AFFIRM, LATIN-MH, PAM-D, RedeAmericas, SHARE: National Institute of Mental Health (NIMH) funded five research hubs aimed at improving the research core for evidence-based mental health interventions, enhancing research skills in global mental health, and providing capacity building (CB) opportunities for early career investigators in LMIC | -Absence of hands-on and focused short training courses | -Inadequate research funding  -Difficulty in accessing a Ph.D. supervisor -Lack of access to international exposure  -Limited skill and commitment to publish papers and difficulty in finding time to work on papers and presentations | -Strong outputs at lower cost and with relatively few additional mentors to rapidly achieve a critical mass of independent scientists able to conduct original research and mentor others. | -Submitted two articles to international journals which are currently in press,  -Won two travel fellowship grants for early career researchers to attend the 2016 and 2017 World Psychiatric Association International Congress  -Completed dissertation project,  - Completed courses for second master’s degree, focused on mental health.  - One award won from Gottingen, Germany, to support the further development of research career.  -One appointment as Professor and Young Researcher at the Institute of Health Sciences |
| 18 | Chelsea M McGuire (2020) Lesotho Mixed-methods design | Early career researchers from family health field recruited from a health facility | Family Medicine Specialty Training Programme (FMSTP): research capacity building was conducted via a blended research curriculum and peer mentorship for 2 years | -Infrastructure gaps, including funding and protected time | NS | NS | -Trainees (n = 8) experienced moderate increases in research confidence that were statistically significant.  -A positive research culture was created, promising for sustainability. |
| 19 | M D Ughasoro et al (2022) Nigeria Quantitative design | Early career health researchers (n=21) from the biomedical, health system and policy, and public health fields, recruited from a research institution | The Nigeria Institute of Medical Research (NIMR) hosted a pioneer 3‑week residential workshop of mentee‑mentor program and Grantsmanship | NS | -Lack of understanding of mentorship process -Lack of capacity for mentoring -Mentor preference  -Lack of freedom of expression -Culture of selfishness  -Lack of formal relationship | NS | NS |
| 20 | Emmanuel Balandya et al (2022) Tanzania  Quantitative design | Early career researchers (n=53); undergraduate students, young faculty members & senior scientists from three partnering universities | Medical Education Partnership Initiative-Junior faculty (MEPI-JF): Fellows of the program received mentorship and research training, research awards and in turn mentored undergraduate students for 4 years. | NS | NS | NS | - Number of fellows increased from 12-24 and mentored graduates from 41-67 second cohort |
| 21 | Mremi et al (2023) Tanzania Quantitative design | Early career researchers (n=12) comprising of junior academicians from a health sciences department of a university | Transforming Health Education in Tanzania (THET) project: senior faculty members mentored junior faculty in three academic institutions for 4 years | NS | -Physical meetings between mentors and mentees were halted due to the emergence of COVID-19.  -Apart from physical meetings, the mentees also suspended some research activities, especially the enrollment of participants and the order of laboratory reagents. | NS | -Most of the mentees (9/12) had at least one published or accepted manuscript out of their respective mentored research projects;  -10 of 12 had registered for a Ph.D. fellowship; and  -7 of 12 had applied for and received research grants for their research program |
