# Supplementary material for: Mentorship in health research institutions in Africa: A systematic review of approaches, benefits, successes, gaps and challenges
Source: PLOS Glob Public Health. 2024 Sep 23;4(9):e0003314. doi: 10.1371/journal.pgph.0003314 (PMC11419371; doi:10.1371/journal.pgph.0003314)
Supplement: S3 Table — (DOCX) [file pgph.0003314.s004.docx]

|  |  | **SCREENING QUESTIONS** | |  | | | | |  | | | | |  | | | | |  |  |
| --- | --- | --- | --- | --- | --- | --- | --- | --- | --- | --- | --- | --- | --- | --- | --- | --- | --- | --- | --- | --- |
| **Author(s)** | **Year** | S1. | S2. | 1.1 | 1.2 | 1.3 | 1.4 | 1.5 | 4.1 | 4.2 | 4.3 | 4.4 | 4.5 | 5.1 | 5.2 | 5.3 | 5.4 | 5.5 | **Score (%)** | **Quality** |
| **Qualitative studies** | |  |  |  |  |  |  |  |  |  |  |  |  |  |  |  |  |  |  |  |
| Daniels et al | 2015 | Y | Y | Y | Y | Y | Y | Y |  |  |  |  |  |  |  |  |  |  | 100 | ***** |
| Ager et al | 2015 | Y | Y | Y | Y | Y | Y | Y |  |  |  |  |  |  |  |  |  |  | 100 | ***** |
| Daniels et al | 2014 | Y | Y | Y | Y | Y | Y | Y |  |  |  |  |  |  |  |  |  |  | 100 | ***** |
| Dartnall et al | 2017 | Y | Y | Y | N | Y | Y | Y |  |  |  |  |  |  |  |  |  |  | 86 | ***** |
| Ezeanolue et al | 2019 | Y | Y | Y | Y | Y | Y | Y |  |  |  |  |  |  |  |  |  |  | 100 | ***** |
| Färnman | 2016 | Y | Y | Y | N | Y | Y | Y |  |  |  |  |  |  |  |  |  |  | 86 | **** |
| Gadhi et al | 2019 | Y | Y | Y | N | Y | Y | Y |  |  |  |  |  |  |  |  |  |  | 86 | **** |
| Langhaug et al | 2020 | Y | Y | Y | N | Y | Y | Y |  |  |  |  |  |  |  |  |  |  | 86 | **** |
| da Silva et al | 2019 | Y | Y | Y | Y | Y | Y | Y |  |  |  |  |  |  |  |  |  |  | 100 | ***** |
| Mda et al | 2013 | Y | Y | Y | Y | Y | Y | Y |  |  |  |  |  |  |  |  |  |  | 100 | ***** |
| **Quantitative studies** | |  |  |  |  |  |  |  |  |  |  |  |  |  |  |  |  |  |  |  |
| [Balandya et al](https://bmcmededuc.biomedcentral.com/articles/10.1186/s12909-021-02611-0#auth-Emmanuel-Balandya-Aff1) | 2021 | Y | Y |  |  |  |  |  | Y | N | Y | Y | Y |  |  |  |  |  | 86 | **** |
| O. Gureje et al | 2019 | Y | Y |  |  |  |  |  | N | N | Y | Y | Y |  |  |  |  |  | 71 | *** |
| Somefun O.D et al | 2021 | Y | Y |  |  |  |  |  | Y | Y | Y | N | Y |  |  |  |  |  | 86 | **** |
| Thomson et al | 2016 | Y | Y |  |  |  |  |  | Y | N | Y | Y | Y |  |  |  |  |  | 86 | **** |
| Torondel et al | 2019 | Y | Y |  |  |  |  |  | Y | Y | Y | Y | Y |  |  |  |  |  | 86 | **** |
| Manabe et al | 2018 | Y | Y |  |  |  |  |  | Y | N | Y | Y | Y |  |  |  |  |  | 86 | **** |
| Ughasoro et al | 2022 | Y | Y |  |  |  |  |  | Y | N | Y | Y | Y |  |  |  |  |  | 86 | **** |
| Balandya et al | 2022 | Y | Y |  |  |  |  |  | Y | N | Y | Y | Y |  |  |  |  |  | 86 | **** |
| Mremi et al | 2023 | Y | Y |  |  |  |  |  | N | N | Y | Y | Y |  |  |  |  |  | 71 | *** |
| **Mixed-methods studies** | |  |  |  |  |  |  |  |  |  |  |  |  |  |  |  |  |  |  |  |
| Hakim, JG et al | 2018 | Y | Y | Y | Y | Y | Y | Y | Y | Y | Y | Y | Y | Y | Y | Y | Y | Y | 100 | ***** |
| McGuire et al | 2020 | Y | Y | Y | Y | Y | Y | Y | Y | Y | Y | N | Y | Y | Y | Y | Y | Y | 94 | **** |
