# Supplementary material for: Mentorship in health research institutions in Africa: A systematic review of approaches, benefits, successes, gaps and challenges
Source: PLOS Glob Public Health. 2024 Sep 23;4(9):e0003314. doi: 10.1371/journal.pgph.0003314 (PMC11419371; doi:10.1371/journal.pgph.0003314)
Supplement: S4 Table — (DOCX) [file pgph.0003314.s005.docx]

| **Author** | **Intervention characteristics** | **Mode of delivery** |
| --- | --- | --- |
| 1. **International (North- South) collaborative programs** | | |
| Daniels et al. 2015 | International HIV/AIDS research collaborations between investigators in the US and low-resource countries primarily designed to provide HIV/AIDS research training to Kenyan medical doctors and to non-medical trainees interested in an epidemiology or basic science research career at the University of Nairobi. | Cross-cultural training program |
| Ager & Zarowsky, 2015 | A collaborative capacity strengthening initiative undertaken by the School of Public Health and Centre for Research in HIV and AIDS of the University of Western Cape (UWC), South Africa in collaboration with US based partners in HIV work. | Global symposia, workshops and mentor-supported research activities |
| Daniels et al. 2014 | Through the AIDS International Training and Research Program, Kenyan HIV/AIDS researchers were supported for an international training program in the US. They then returned to their home country after completing the program to apply this new combined local–international knowledge to address health issues through research. | Cross-cultural training program |
| Farnman et al. 2016 | 4-year North-South collaboration that used innovative educational technologies to strengthen health research across Africa and Asia focusing on postgraduate, doctoral and postdoctoral training. Cutting-edge online courses were developed, as well as blended learning modules and joint programmes that enabled training of researchers in LMICs who might not otherwise have access to such material. The program also worked at institutional level to strengthen education services, financial and administrative research management, research uptake capacity and network building. | Postgraduate, doctoral and postdoctoral training with additional online courses blended learning modules and joint programmes. |
| Gureje et al (2019) | A 4-year North-South multinational collaboration bringing together partner institutions from 5 African countries with researchers from USA and the UK in partnership with governments and NGOs to create an infrastructure to develop research capacity. | Workshops and fellowships targeting specific skill-sets in research. |
| Hakim et al (2018) | A 5-year medical education and research strengthening north-south collaborative initiative meant to offer a range of medical education and research capacity-focused programs including faculty development, research support, mentored scholars, visiting professors, community-based education, information and technology support, crosscutting curricula, and collaboration with partner universities and the ministries of health and education. | Workshops facilitated by local and partner universities. |
| 1. **Regional and in-country collaborations** | | |
| Dartnall et al. 2017 | For a duration of 3 years, sexual Violence Researchers from Kenya, Uganda, and Tanzania were provided with intensive mentoring and technical advice in development or adaptation and conduct of preliminary proof of concept testing of violence against women and violence against children primary prevention interventions. | South to south collaboration involving technical advisors from south Africa supporting researchers from three countries through face to face meetings and partnerships in research projects. |
| Ezeanolue et al. 2019 | Nigeria Implementation Science Alliance (NISA): Programme involved members of NISA involved in facilitating collaboration among partners, enhancing implementation research in Nigeria and the sub-Saharan region, and identifying feasible, culturally appropriate strategies to improve public health through research and participated in the 2017 NISA scientific conference. | An in-country partners alliance system for implementation research. |
| Gandhi et al. 2019 | A series of regional, 2-day intensive mentorship workshops were conducted for a period of 4 years to train mid- and senior-level investigators conducting public health, clinical, and basic science research across multiple academic institutions in LMICs to be more effective mentors. | Research training through workshops |
| da Silva et al, 2019 | National Institute of Mental Health (NIMH) funded five research hubs (AFFIRM, LATIN-MH, PAM-D, RedeAmericas, SHARE) aimed at improving the research core for evidence-based mental health interventions, enhancing research skills in global mental health, and providing capacity building (CB) opportunities for early career investigators in LMIC | Global networks and infrastructure development through multinational research hubs. |
| Mremi et al, 2023 | Transforming Health Education in Tanzania (THET) project: Senior faculty members mentored junior faculty in three academic institutions for 4 years. | Research skills support by senior researchers |
| 1. **Specialized capacity programs** | | |
| Balandya et al, 2021 | Through the 5-year Transforming Health Professions Education in Tanzania (THET)-project, young peers received mentorship from senior researchers from a consortium through mentored research awards and research training, and in turn provided reciprocal peer-to-peer mentorship as well as mentorship to undergraduate students. | Mentored research awards and research training |
| Somefun et al. 2021 | Local research capacity strengthening programme delivered through PhD training fellowships. CARTA trains Africa-based doctoral fellows who admitted in cohorts with a goal of creating a network of locally trained but globally recognised scholars. | PhD training fellowships |
| Mda, 2013 | Growing Researchers: senior staff at two research councils mentored Master’s and doctoral students: South African Medical Research Council (MR) and Human Sciences Research Council (HSRC). | Research skills support by senior researchers |
| Thomson et al. 2016 | A 6-week deliverable-driven survey analysis training based in Rwanda was conducted to strengthen skills of five local research leaders, 15 statisticians, and a PhD candidate | Skills support through training with focus on analysis |
| Torondel et al. 2019 | Sanitation and Hygiene Applied Research for Equity (SHARE): Delivered via (1) structured mentoring integrated into the research, administration, financial management and communication activities; (2) specific training to address immediate gaps in skills; and (3) a PhD programme designed to build lasting research capacity within LMIC institutions (including non-governmental organisations and universities) for 5 years | Institutional systems support, trainings for skills support and PhD programmes. |
| Yukari, 2018 | MEPIMESAU: The program provided administrative support, paid tuition fees, tools (space, equipment, research money), skills (short research courses on study design, biostatistics, manuscript and grant writing), and infrastructure (finance, grants management support, and lab infrastructure) to early career researchers for 5 years | Infrastructural support and skills development training. |
| Langhaug, 2020 | African Mental Health Research Initiative (AMARI): 48 researcher fellows at Master's PhD and post-doc levels were recruited and trained with the intent of equipping them with the necessary research, teaching and leadership skills to build a viable and sustainable research network in the African region. | Training programs for specific disciplines e.g. focus on mental health research |
| Chelsea, 2020 | Family Medicine Specialty Training Programme (FMSTP): research capacity building was conducted via a blended research curriculum and peer mentorship for 2 years | Curriculum development and skills support by senior researchers. |
| Balandya et al, 2022 | Medical Education Partnership Initiative-Junior faculty (MEPI-JF): Fellows of the program received mentorship and research training, research awards and in turn mentored undergraduate students for 4 years. | Research training and skills support |
