# Supplementary material for: Mentorship in health research institutions in Africa: A systematic review of approaches, benefits, successes, gaps and challenges
Source: PLOS Glob Public Health. 2024 Sep 23;4(9):e0003314. doi: 10.1371/journal.pgph.0003314 (PMC11419371; doi:10.1371/journal.pgph.0003314)
Supplement: S5 Table — (DOCX) [file pgph.0003314.s006.docx]

| AESA | - | Alliance for Accelerating Excellence in Africa |
| --- | --- | --- |
| AFFIRM | - | Africa focus on intervention research for mental health |
| AITRP | - | AIDS International Training and Research Program |
| AJOL | - | African journals online |
| AMARI | - | African Mental Health Research Initiative |
| ARCADE | - | African/Asian Regional Capacity Development |
| AREF | - | African Research Excellent Fund |
| APHRC | - | African Population and Health Research Center |
| CARI | - | Coalition for Research and Innovation |
| CARTA | - | Consortium for Advanced Research Training in Africa |
| DHS | - | Demographic Health Survey |
| DOAJ | - | Directory of open access journals |
| EMBASE | - | Excerpta medical database |
| JSTOR | - | Journal storage |
| LATIN – MH | - | Latin America Treatment and Innovation Network in Mental Health |
| LMIC | - | Low- and middle-income country |
| MMAT | - | Mixed Methods Appraisal Tool |
| MeSH | - | Medical Subject Headings |
| MEPI-MESAU | - | Medical Education Partnership Initiative – Medical Education for Equitable Services for All Ugandans |
| NACA | - | National Agency for the Control of AIDS |
| NECTAR | - | Novel Education Clinical Trainees and Researchers |
| NIMH | - | National Institute of Mental Health |
| NISA | - | Nigeria Implementation Science Alliance |
| PAM-D | - | Partnership for mental health development |
| PICOS | - | Population, intervention, comparison, outcome, and setting |
| PRISMA | - | Preferred Reporting Items for Systematic Reviews and Meta-Analyses. |
| PROSPERO | - | International Prospective Register of Systematic Reviews |
| RedeAmericas | - | Regional Network for Mental Health Research in Latin America |
| RRCS | - | Research and Related Capacity Strengthening |
| SHARE | - | Sanitation and Hygiene Applied Research for Equity |
| THET | - | Transforming Health Professions Education in Tanzania |
| UWC | - | University of Western Cape |
