# Supplementary material for: Mentorship in health research institutions in Africa: A systematic review of approaches, benefits, successes, gaps and challenges
Source: PLOS Glob Public Health. 2024 Sep 23;4(9):e0003314. doi: 10.1371/journal.pgph.0003314 (PMC11419371; doi:10.1371/journal.pgph.0003314)
Supplement: Attachment — Submitted filename: Point by point response.docx [file pgph.0003314.s009.docx]

**Point by point response to reviewer and editorial comments**

**Editorial comments**

Thank you for this interesting paper. The reviewer has some suggestions for improvement which I believe will strengthen your paper. I realise that re-running the review with individual African country names may seem to be a large task - but I would urge you to run a check to see that nothing has been missed. You can then update your methods to assure readers that this was done. If you find papers that were missed, they can be added in and you can explain in your methods that you ran a country search separately to ensure that no papers were missed.

**Response. We thank you for considering our manuscript for review. We received valuable comments from the reviewers and have addressed all of them. We agree that re-running the review with the individual African country names would be a huge task. However, we have performed additional searches at the regional level, replacing the names of each region with the country names. This has simplified the search process. We have responded to the reviewer's comments in response to comment #2. Our new text reads as follows:**

**“To ensure that we retrieved articles from each country, we substituted the names of the study setting with the country-specific names. For example, to retrieve articles from East Africa, we replaced "East Africa" with Uganda, Kenya, Tanzania, Burundi, and Rwanda.”**

**Review Comments to the Author

Reviewer #1**

1. This is a very well written systematic review of the landscape of mentorship across African institutions. The authors have described their systematic approach to the study, which was registered on PROSPERO and follows the PRISMA guidelines and the PICO approach applied using MMAT for reviews of mixed methods studies. The review outlines the key benefits and challenges related to different mentorship models for capacity strengthening in health research in Africa. I only have a couple of minor comments on the paper:

**Response. Thank you for providing us with insightful comments that have improved the quality of the manuscript.**

2. The initial search term do not include lists of individual country names and I wonder if this has led to missing some initiatives from the study

**Response. Thank you for this comment. We performed the search for each country but this was done by replacing the study setting, for example “East Africa” with each of the county specific names. We realized that inserting a search strategy with the names of each county would make the search strategy so huge so we opted to maintain the names of each region. In the revised manuscript, we have provided further details regarding how the article retrieval was conducted at country level and the text read as follows:**

**“To ensure that we retrieved articles from each country, we substituted the names of the study setting with the country-specific names. For example, to retrieve articles from East Africa, we replaced "East Africa" with Uganda, Kenya, Tanzania, Burundi, and Rwanda.”**

**We have also performed additional search for other articles that might have been published to date.**

3. The authors explicitly state that the review will consider effectiveness but do not provide a clear definition of what effectiveness means in this context. Inclusion of this would substantially enhance the review.

**Response. We have provided a clear definition of the effectiveness of mentorship in the concluding section of the introduction. The new text reads as follows:**

**“Mentorship was considered effective if it resulted in early and mid-level career researchers taking up leadership roles and positions in research and academia, fostering a collaborative research environment, contributing to research outputs such as publications, and strengthening the skills of early and mid-level career researchers.”**

4. This is perhaps beyond the scope of this review but I would be interested to see how capacity strengthening impacted African researchers by gender, I wonder if the authors might comment on this at least?

**Response. Thank you for this valuable comment. In this review, only 7 studies reported gender differences at enrolment into the mentorship intervention. However, none of the included studies reported the impact of capacity strengthening by gender. Therefore, it was, impractical to report the impact of mentorship by gender.**

5. I also wonder whether the authors might be able to comment in the conclusions on which approaches were considered most successful in terms of outputs based on mentorship approaches - this would substantially increase the importance of this paper
   These are simply recommendations for minor revisions, to move towards greater interpretation of the findings, but the paper is well structured, clearly thought through and very well written overall.

**Response. We are grateful for this comment. While we agree that providing information regarding the most successful mentorship approach is critical to improving the quality of the manuscript, we do not have the data as it was not initially considered as part of the review. We have included this concern as one of the review limitations and suggested that future reviews should focus on the topic. The new text in the limitations section reads as follows:**

**“Additionally, we did not synthesize data regarding the most successful mentorship approaches in terms of the outputs so this topic should be explored in future reviews”.**
